# Structural basis of the high thermal stability of the histone-like HU protein from the mollicute *Spiroplasma melliferum* KC3

**DOI:** 10.1038/srep36366

**Published:** 2016-11-03

**Authors:** Konstantin M. Boyko, Tatiana V. Rakitina, Dmitry A. Korzhenevskiy, Anna V. Vlaskina, Yuliya K. Agapova, Dmitry E. Kamashev, Sergey Y. Kleymenov, Vladimir O. Popov

**Affiliations:** 1 Kurchatov Complex of NBICS-Technologies, National Research Center “Kurchatov Institute”, Akad. Kurchatova pl., 1, Moscow, 123182, Russian Federation; 2 Bach Institute of Biochemistry, Research Center of Biotechnology of the Russian Academy of Sciences, Leninsky Prospekt. 33, bld. 2, 119071, Moscow, Russian Federation; 3 Laboratory of Hormonal Regulation Proteins, M.M. Shemyakin–Yu.A. Ovchinnikov Institute of Bioorganic Chemistry, Russian Academy of Sciences, ul. Miklukho-Maklaya, 16/10, Moscow, 117997, Russian Federation; 4 Koltzov Institute of Developmental Biology, Russian Academy of Sciences. ul. Vavilova, 26, Moscow, 119334, Russian Federation

## Abstract

The three-dimensional structure of the histone-like HU protein from the mycoplasma *Spiroplasma melliferum* KC3 (HUSpm) was determined at 1.4 Å resolution, and the thermal stability of the protein was evaluated by differential scanning calorimetry. A detailed analysis revealed that the three-dimensional structure of the HUSpm dimer is similar to that of its bacterial homologues but is characterized by stronger hydrophobic interactions at the dimer interface. This HUSpm dimer interface lacks salt bridges but is stabilized by a larger number of hydrogen bonds. According to the DSC data, HUSpm has a high denaturation temperature, comparable to that of HU proteins from thermophilic bacteria. To elucidate the structural basis of HUSpm thermal stability, we identified amino acid residues potentially responsible for this property and modified them by site-directed mutagenesis. A comparative analysis of the melting curves of mutant and wild-type HUSpm revealed the motifs that play a key role in protein thermal stability: non-conserved phenylalanine residues in the hydrophobic core, an additional hydrophobic loop at the N-terminal region of the protein, the absence of the internal cavity present at the dimer interface of some HU proteins, and the presence of additional hydrogen bonds between the monomers that are missing in homologous proteins.

HU proteins are the most abundant DNA-binding proteins in prokaryotic organisms and play an essential role in processes of DNA replication, repair, and recombination^1^. HU proteins, as well as IHF, H-NS, and others, belong to the class of nucleoid-associated proteins (NAPs), which bind to the DNA minor groove either specifically (IHF) or nonspecifically (HU), thus inducing and/or stabilizing DNA bending and supercoiling^2^. The pleiotropic role of HU proteins in bacterial cells is due to their involvement in DNA compaction in the bacterial nucleoid and the regulation of DNA transactions, including transcription^3^,^4^. The NAP repertoire varies between bacteria, and other non-HU NAPs are able to perform HU functions to a certain extent^5^. *E. coli* with a genetic deletion of HU is viable but has multiple growth defects under altered conditions, including high and low temperature, high osmolality, UV irradiation, or nutrient deficiency^4^,^6^,^7^. In bacteria such as *B. subtilis* and *M. genitalium*, where only HU proteins serve the function of NAPs, the genetic deletion of this protein is lethal^8^,^9^,^10^. Moreover, it has been recently found that small-molecule inhibitors of the *M. tuberculosis* HU protein predicted by structure-based design have antibacterial activity^11^. Therefore, HU proteins can be considered promising targets for the development of new therapies for infectious diseases.

HU proteins are small (approximately 90 amino acids per monomer) basic dimeric proteins that are annotated in the majority of bacteria with sequenced genomes. In most bacteria, HU protein is a dimer of identical subunits, though heterodimeric HU is characteristic of enterobacteria, including *E. coli*, *S. typhimurium*, and *S. marcescens*^12^.

Three-dimensional structures of a number of HU proteins, their mutants and complexes with DNA have been solved, among which are those from *E. coli*^13^,^14^, the cyanobacterium *Anabaena*^15^, the pathogenic bacteria *S. aureus*^16^, *B. anthraci*, *B. burgdorferi*^17^, and *M. tuberculosis*^11^, and the thermophilic bacteria *B. stearothermophilus*^18^ and *T. maritima*^19^. All HU dimers feature a compact architecture, including several intertwined α-helices, two three-stranded β-sheets, and two disordered arms that are flexible in the absence of DNA^19^. Due to the existence of HU homologues possessing moderate^14^,^20^,^21^,^22^ or high^19^,^20^,^22^,^23^,^24^,^25^,^26^ thermal stability, the small size of the proteins, and the similarity of the three-dimensional structures, HU proteins may serve as a convenient model for investigating the structural basis of thermal stability. It has been shown that thermal denaturation of HU proteins usually occurs through dissociation of the dimer into denatured (unfolded) monomers^18^,^22^,^25^ with the only known exception being the *E. coli* HU protein, which undergoes a two-step denaturation described by the dimer – dimeric intermediate – denatured monomers model^14^. In this case, the dimeric intermediate is suggested to facilitate the *de novo* formation of heterodimers, which may play an essential biological role^14^.

Mollicutes, commonly referred to as mycoplasmas, are the smallest known microorganisms. They are characterized by the absence of a cell wall, a parasitic lifestyle, and reduced genome size^27^,^28^. HU proteins from *Acholeplasma laidlawii* and *Mycoplasma gallisepticum* have been characterized^29^,^30^, but structural data for HU proteins from mycoplasmas have long been lacking. Recently, the HU protein from *Spiroplasma melliferum* KC3, an insect parasite infecting honey bees^31^, was produced in *E. coli*, purified, and crystallized, and the three-dimensional structure of this protein, referred as HUSpm, was solved at high resolution^32^.

Here, we report detailed analysis of the three-dimensional structure of HUSpm and the results of a comparative study on the thermal stability of wild-type and mutant HUSpm by differential scanning microcalorimetry (DSC). We show that although HUSpm originates from a mesophilic organism, it possesses a unique thermal stability comparable to that of HU proteins from thermophiles. The structural motifs responsible for this property of HUSpm were primarily identified based on a comprehensive comparison of the three-dimensional structures of HUSpm and homologous proteins exhibiting different thermal stability. The important role of the identified amino acid residues for the thermal stability of HUSpm was further confirmed by site-directed mutagenesis followed by DSC analysis of the mutant proteins.

## Results

### Overall protein structure

**Figure 1:**
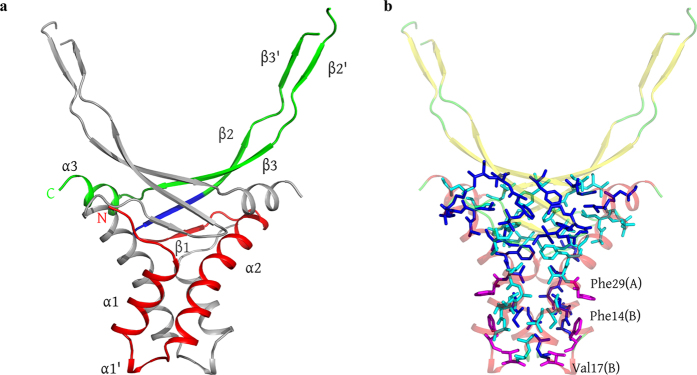
HUSpm dimer. (**a**) Ribbon structure of the HUSpm dimer coloured by the canonical domain structure: the HTH domain in red, the DBD domain in green, and the DS region in blue (see comments in the text). The N- and C-termini for one subunit are indicated. One monomer is coloured in grey for clarity. (**b**) The residues involved in the formation of the hydrophobic core of the HUSpm dimer are shown in blue and cyan for two monomers, respectively. The orientation of the molecule is the same as in (**а**). The non-conserved Phe14, Phe29, and Val17 residues of both monomers are in magenta. For the reasons of clarity, the ribbon model of the dimer is semitransparent.

The monomer of recombinant HUSpm consists of 95 residues including an additional Gly-His dipeptide preceding the N-terminal Met, which was introduced by the cloning procedure^32^. The three-dimensional structure of the HUSpm monomer shown in Fig. 1 is similar to those of known HU proteins (Supplementary Table S1, Figure S1). The monomer is composed of the following three canonical components: a helix-turn-helix (HTH) domain, a dimerization signal (DS) consisting of residues 48–52, and a flexible extended “arm” region — the DNA binding domain (DBD) region — responsible for DNA binding (Fig. 1a).

**Figure 2:**
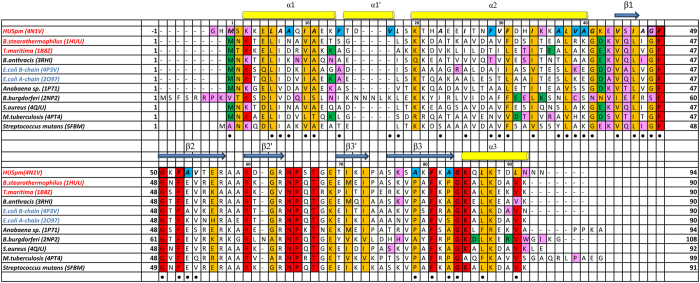
Multiple sequence alignment of HUSpm and HU proteins with known three-dimensional structures. The PDB code is given in parentheses after the name of the organism. The name of the organism is red if its HU protein has high thermal stability and blue if its HU protein has low thermal stability. The HUSpm secondary structure elements are indicated. The residues involved in the formation of the DS region are in black frames. Residues involved in the formation of the hydrophobic core of HUSpm are marked with a black circle. Non-homologous residues of the HUSpm hydrophobic core are in blue. Residues that form hydrogen bonds in the dimers of HU proteins are in magenta and residues that form salt bridges in the dimers of HU proteins are in green. The N-terminal Met residue in all HU proteins, except for HUSpm and the HU protein from *B. burgdorferi*, forms both a salt bridge and a hydrogen bond.

The HTH domain of HUSpm consists of two α-helices denoted as α1 (residues 3–13) and α2 (residues 20–40) linked to each other by a flexible loop (residues 14–19) containing an additional short helix α1′ (residues 15–17), which is missing in the known structures of HU proteins (the role of this helix is discussed below). The DBD region comprises three antiparallel β-strands denoted as β1 (residues 44–46), β2 (residues 50–58), and β3 (residues 76–83); the extended arm consisting of two antiparallel β-strands denoted as β2′ (residues 60–63) and β3′ (residues 70–73); and the C-terminal α-helix α3 (residues 85–92) (Fig. 2).

### Dimer formation

As is seen in all known HU proteins, which are either homo- or heterodimers, the functional unit of HUSpm is a homodimer composed of two equivalent monomers (one monomer per asymmetric unit) related by a crystallographic twofold axis (Fig. 1a). An analysis of the residues involved in the interface revealed the 14 hydrogen bonds listed in Table 1; no salt bridges are present. In recombinant HUSpm, the N-terminal glycine residue Gly(−1) is also involved in stabilization of the dimer (Table 1). It is known that dimers of HU proteins have a large hydrophobic core at the centre of the dimer interface. In HUSpm, this core includes 35 hydrophobic residues from each monomer (Fig. 1b).

### Thermal stability of HUSpm by DSC

**Figure 3:**
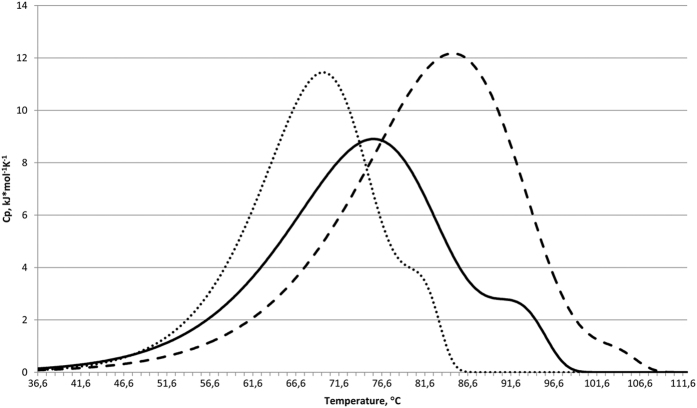
The effects of protein concentration and ionic strength on the temperature dependence of the excess heat capacity of HUSpm denaturation measured by DSC. The plots are given by a solid line for 4.5 mg/ml protein in 0.2 M NaCl, by a dashed line for 4.5 mg/ml protein in 1 M NaCl, and by a dotted line for 1 mg/ml protein in 0.2 M NaCl.

The thermal denaturation of HUSpm was studied using differential scanning calorimetry (DSC) under conditions similar to those reported for the *E. coli* HU protein^14^ (Table 2). The DSC curve (Fig. 3) shows the presence of two thermal transitions with maxima at 75.5 and 92.2 °С when analysed at a concentration of 4.5 mg/ml in the presence of 0.2 M NaCl (Table 2). A similar melting curve profile of *E. coli* HU homo- and heterodimers had previously been observed i^14^. The DSC curves of the latter proteins show two melting peaks, the first of which was assigned to the partial melting of some α-helices, without loss of the dimeric state, followed by the dissociation to denatured monomers (the second melting peak).

A comparison of the thermal denaturation profile of HUSpm with that of HU proteins from mesophilic and thermophilic organisms (Table 2) showed that HUSpm has an unusually high thermal stability among the mesophilic proteins, which is comparable to that of the proteins isolated from the thermophilic bacteria *B. stearothermophilus* and *T. maritima*.

An investigation into the effect of ionic strength on the denaturation of HUSpm showed that an increase in NaCl concentration to 1 M causes a shift of the denaturation peaks to higher temperatures (84.8 and 103.3 °С, respectively; see Fig. 3), which is indicative of the significant contribution of hydrophobic interactions to the stability of the dimer^14^ and is consistent with results obtained for other proteins of this class (Table 2).

Decreasing the HUSpm concentration to 1 mg/ml causes a shift of both denaturation peaks in the opposite direction to lower temperatures (69.9 and 81.0 °С, respectively; Fig. 3). This is also consistent with the results obtained earlier for *B. subtilis* and *T. volcanium* HU proteins, which undergo one-step denaturation (Table 2). In the DSC curves of *E. coli* HU homo- and heterodimers, only the position of the second (higher-temperature) peak is protein concentration dependent, which suggests that the dimer dissociation takes place immediately before denaturation of the high-temperature domains^14^. In contrast, both peaks in the DSC curve of HUSpm exhibit a dependence on the protein concentration, which implies that the denaturation of either calorimetric domain begins after the dimer dissociation.

### Structural basis of thermal stability of HUSpm

To reveal the structural basis of thermal stability of HUSpm, we compared interactions (*e.g*., hydrophobic interactions, hydrogen bonds, and salt bridges) at the dimer interfaces of HU proteins with known three-dimensional structures and well-characterized thermal stability (Table 3).

Unlike other HU proteins with known three-dimensional structures, there are no salt bridges across the dimer interface in HUSpm, although a larger number of hydrogen bonds are formed compared to other HU proteins (Table 3). In particular, the presence of a Gly-His dipeptide at the N-terminus of the recombinant HUSpm^32^ leads to the loss of two salt bridges between the main-chain nitrogen atom of Met1 and residues of the β1 strand of another monomer. Instead, four additional hydrogen bonds involving the Gly(−1) and Met1 residues of each monomer are formed (Table 1). The buried surface area of HUSpm with Gly-His at the N-terminus calculated by PDBePISA is approximately 170 Å^2^ larger than that of HUSpm without the N-terminal dipeptide. Therefore, the N-terminal fragment of HUSpm contributes to the stabilization of the HUSpm dimer.

An interesting feature of HUSpm is the presence of hydrogen bonds between the β3 and α3 regions (Lys77-Asn92 and *vice versa*) and between the α2 helix and the loop that links strands β1 and β2 (Gly48-Lys35 and *vice versa*) (Table 1, Fig. 2). In the structures of other HU proteins with known thermal stabilities, these bonds are not observed. The presence of these bonds may also contribute to the stabilization of the dimeric structure of HUSpm.

An analysis of the dimer interface area (i.e., the buried surface area of the monomer upon the dimer formation) in HU proteins demonstrates that this parameter has the maximum value in HUSpm (Table 3). However, the overall percentage surface area is approximately equal for all HU proteins. The number of amino acid residues comprising the interface (including hydrophobic residues) is also approximately the same in all HU proteins. The strength of the hydrophobic interactions, as estimated from the solvation free energy gain upon the formation of the dimer (calculated by PDBePISA), is higher for HUSpm compared to other HU proteins (Table 3). The difference in the strength of hydrophobic interactions in the dimers of HU proteins is related to the size and mutual arrangement of hydrophobic residues rather than by their quantity.

HUSpm contains two non-conserved phenylalanine residues, Phe14 and Phe29 (Figs 1b and 2). In the structurally characterized HU proteins, these positions are occupied by residues with a short and not necessarily hydrophobic side chain. In HUSpm, both non-conserved phenylalanine residues are located at the hydrophobic dimer interface. An analysis of the three-dimensional structure of HUSpm demonstrates that residue Phe14 of one monomer is located near Phe29 of the other monomer (Fig. 1b), which is favourable for the strengthening of the hydrophobic contact. The hydrophobic core of the HUSpm dimer may be additionally stabilized by the non-conserved residue Val17 (Fig. 2), which is located on the small helix α1′ in the vicinity of Phe14 and Leu18 and is also involved in the formation of the hydrophobic dimer interface of HUSpm (Fig. 1b). There is also one semi-conserved Phe at position 31 in the hydrophobic core of HUSpm, which may also stabilize the hydrophobic interface.

A comparative analysis of the three-dimensional structure of HUSpm revealed amino acid residues potentially responsible for high thermal stability of the protein, including residues that strengthen the dimeric hydrophobic contact: the two non-conserved phenylalanine residues Phe14 and 29, and the semi-conserved Phe31 and Val17 of the additional hydrophobic loop at the N-terminal region of the protein. Additionally, Lys35 and Asn92, which are missing in homologous proteins, are involved in the formation of additional hydrogen bonds between the monomers.

### Comparative DSC of HUSpm mutants

To examine the effects of the above residues on the thermal stability of HUSpm, we produced and tested a number of point mutants (listed in Supplementary Table S2). All mutants had a Gly-His dipeptide at the N-terminus and form stable dimers, which was confirmed by size-exclusion chromatography. The mutants also demonstrate DNA-binding properties similar to those of wild-type (WT) HUSpm (data not shown).

**Figure 4:**
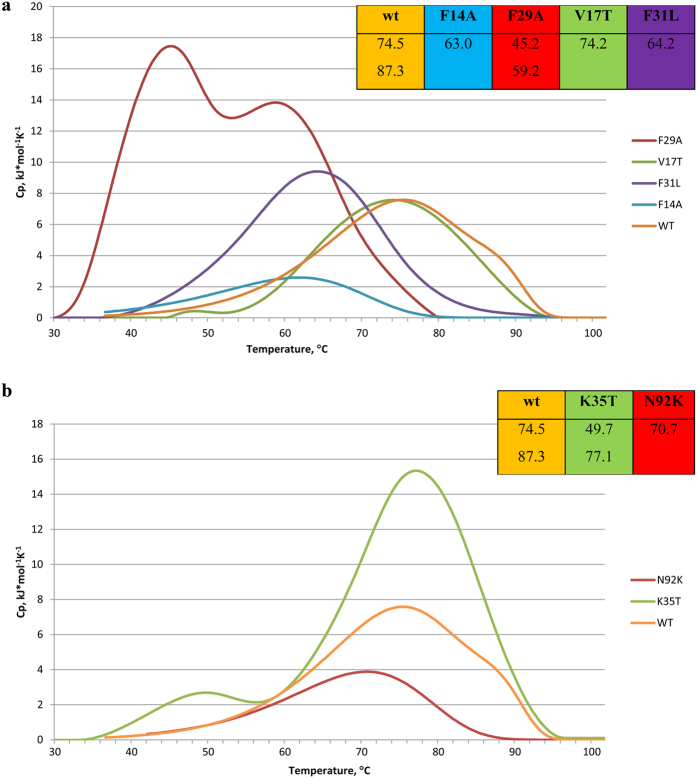
Temperature dependence of the excess heat capacity of denaturation measured by DSC for wild-type HUSpm and its point mutants. In all cases, the protein concentration is 2.0 mg/ml and the NaCl concentration is 0.2 M. Temperatures of the melting peaks are shown in the corresponding insertions. (**a**) Effects of mutations on the non-conserved or semi-conserved hydrophobic residues. (**b**) Effects of mutations on the non-conserved residues involved in hydrogen bonding.

The thermal denaturation of WT HUSpm and all mutants was studied by DSC at a protein concentration of 2.0 mg/ml in 10 mM sodium phosphate buffer, pH 7.4, containing 0.2 M NaCl. A comparative analysis of thermal denaturation of all six mutants and WT HUSpm (reference control) is shown in Fig. 4 and Supplementary Table S2. Figure 4a illustrates the effects of mutations of the residues that potentially strengthen the hydrophobic dimer contact in HUSpm; Fig. 4b shows the effects from eliminating non-canonical hydrogen bonds between HUSpm monomers.

The melting curve of WT HUSpm measured in the experimental conditions given above shows two denaturation peaks at 74.5 and 87.3 °С (Fig. 4 and Supplementary Table S2). Mutations are reflected in the denaturation profile of the protein, resulting in such changes as the shift of the denaturation peak in the melting curves and, in some cases, the disappearance of one of the two peaks typical of WT HUSpm.

A single peak observed in the melting curves of some mutants may be attributed to the superposition of two closely spaced peaks. However, more research is required to confirm this suggestion. To assess the relative thermal stability of the point mutants that display a single denaturation peak, we compared the positions of their peaks with that of the lower-temperature peak in the melting curve of the WT protein, taking into account that the dissociation of the HUSpm dimer takes place immediately before the first thermal transition.

Mutations of non-conserved and semi-conserved phenylalanine residues (Phe14, Phe29, and Phe31) cause a decrease in the melting point, which is especially substantial for the Phe29Ala mutation, for which the shift was 29 and 27 °C for the low- and high-temperature peaks, respectively. In the case of the Phe14Ala mutation, only one melting peak, shifted to lower temperature by 11 °C, was observed in the denaturation curve. A similar effect (i.e., a 10 °C decrease in the melting point and one peak in the denaturation curve) was observed upon mutation of the Phe31 residue. In contrast, the disruption of the additional α1′ helix, which is involved in the formation of a hydrophobic contact between the monomers, by mutating Val17 to the polar residue Thr had only a slight effect on the protein thermal stability (0.3 °С decrease in the melting point, Fig. 4A, Supplementary Table S2).

Mutations in the two non-conserved residues, Asn92 and Lys35, which are involved in the formation of unique Lys35-Gly48 and Asn92-Lys77 hydrogen bonds between the HUSpm monomers (Table 1, Fig. 2), cause the melting point to decrease. The Lys35Thr mutation causes shifts of approximately 24 °C and 10 °C for the low- and high-temperature peaks, respectively. In the denaturation curve for the Asn92Lys mutant, a single peak was observed at a temperature of approximately 4 °C lower than the first peak in the denaturation curve of wild-type HUSpm (Fig. 4b, Supplementary Table S2).

## Discussion

In the present work, we analysed the three-dimensional structure of the HU protein from the insect parasite mycoplasma S. *melliferum* determined at 1.4 Å resolution and found that it has unusual thermotolerance comparable to that of HU homologues from thermophilic bacteria. The physiological role of such high thermal stability of HUSpm is unclear. However, the residues responsible for the high thermal stability were revealed by a comprehensive structural analysis of the protein and confirmed by comparative DSC of the wild-type and mutant forms of HUSpm.

Taking into account that HU proteins exist as stable dimers and their thermal denaturation involves the dissociation to denatured monomers, we suggest that the contacts at the dimer interface are the keystone of the high thermal stability of HUSpm. Previous studies of the thermal denaturation of HU proteins from *E. coli*, *B. subtilis*, and *T. volcanium* showed that the melting point increases with increasing protein concentration and increasing ionic strength of the solution, which suggests a substantial contribution of hydrophobic interactions to the stability of HU dimers^14^,^21^,^25^ (Table 2). In addition to hydrophobic interactions at the dimer interfaces, the role of other structural factors (e.g., hydrogen bonds and salt bridges) in the thermal stability of HU proteins has also been discussed in a number of reports^14^,^19^,^20^,^21^,^22^,^23^,^24^,^25^,^26^. For example, it was shown that residues Gly15, Glu34, and Val42 (numbering corresponds to the sequence of the *T. maritima* protein) are responsible for high thermal stability of HU proteins from *T. maritima* and *B. stearothermophilus*^20^,^22^,^24^,^26^. The replacement of Gly15 with Glu led to a substantial decrease in the thermal stability of the protein due to destabilization of the HTH domain structure^19^,^24^. In the *T. maritima* HU protein, Glu34 forms a salt bridge with Lys13 and Val42 is involved in the formation of the hydrophobic core, the replacement of the latter residue by a residue with a larger side chain (Ile) destabilized the dimer^19^,^26^. However, in HUSpm, only Val44 (Val42 according to the sequence of *T. maritima*) is involved in the formation of the hydrophobic dimer contact, whereas Thr15 (Gly15 according to the sequence of *T. maritima*) and Lys36 (Glu34 according to the sequence of *T. maritima*) do not form any bonds with the residues of other monomers (Fig. 2). These data suggest that other factors are responsible for high thermal stability of HUSpm.

A comparison of the three-dimensional structures of HU proteins from thermophilic and mesophilic bacteria showed that the fold of HUSpm appears to be typical of this class of proteins, but it is distinguished by stronger hydrophobic interactions and an increased number of hydrogen bonds at the dimer interface, but no salt bridges are present across this interface (Tables 1 and 3). The mutations of Asn92 and Lys35, which are involved in the formation of unique hydrogen bonds between HUSpm monomers, confirmed the impact of these residues on protein thermostability (Fig. 4b, Supplementary Table S2).

An analysis of the amino acid sequences showed that HUSpm contains six Phe residues, compared to the three to four Phe residues in other HU proteins (Fig. 2). It should be noted that all phenylalanine residues in HU proteins are involved in the formation of the hydrophobic core at the dimer interface^22^. In HUSpm, as we mentioned above, the non-conserved Phe14 and Val17 from one monomer are located near the non-conserved Phe29 of the other monomer, stabilizing the hydrophobic core of the dimer (Fig. 1b). It was previously shown that Ala27 in the HU protein from *B*. s*tearothermophilus*, located in the same position as Phe29 in HUSpm, plays an important role in high thermal stability of the protein by creating a more compact hydrophobic dimeric core; the replacement of this residue with Ser resulted in a 5 °C decrease in the thermal stability of the protein^23^. The thermal stability of the mutants Phe14Ala, Phe29Ala and Val17Thr was also analysed, and the key roles of Phe29 and Phe14 in HUSpm thermotolerance was established (Fig. 4a, Supplementary Table S2).

**Figure 5:**
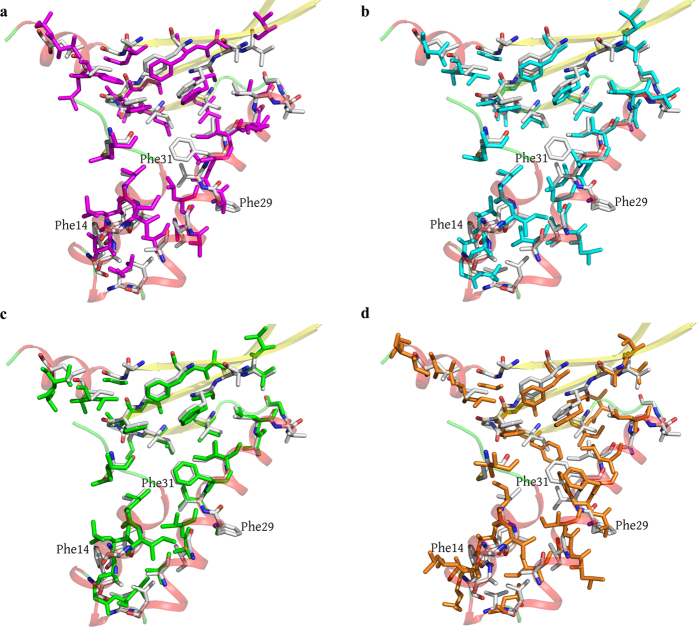
Superposition of the residues involved in the formation of the hydrophobic core in the monomers of HUSpm (shown in gray) and (**а**) *Anabaena* PCC7120 HU protein (pink), (**b**) *T. maritima* HU protein (cyan), (**c**) *B. stearothermophilus* HU protein (green), and (**d**) *B. burgdorferi* HU protein (orange). Only hydrophobic residues are shown. For the reasons of clarity, the secondary structure elements of HUSpm (coloured as in Fig. 2a) are semitransparent.

An intriguing feature of the HU dimers is the presence of an internal cavity with a size of approximately 100 Å^3^ located in the hydrophobic region between the monomers of some proteins. This cavity was found in the dimers of the HU proteins from the cyanobacteria *Anabaena* PCC7120^15^ and *T. maritima*, but it is absent in the *B. stearothermophilus* HU protein (Fig. 5a–c). In HUSpm, this cavity is also absent, which may be attributed to the amino acid substitution at position 31. In HUSpm, like in the *B. stearothermophilus* protein, this position is occupied by Phe, whereas Leu, with a smaller side chain, is present at this position in the proteins from *Anabaena* PCC7120 and *T. maritima* (Fig. 2). It is interesting that the *B. burgdorferi* HU protein also contains phenylalanine at the position analogous to Phe31 in HUSpm. However, in the *B. burgdorferi* HU protein, the side group of this Phe residue is shifted from the centre of the dimer by 2.8 Å (the distance between the CZ atoms) with respect to the positions of Phe in the *S. melliferum* and *B. stearothermophilus* HU proteins (Fig. 5d), resulting in the presence of a cavity in the latter case. The role of this cavity is unclear. Apparently, the absence of this cavity may stabilize the hydrophobic interface in HUSpm due to tighter contacts between the Phe31 of both monomers; mutagenesis confirmed the impact of Phe31 on the thermal stability of HUSpm (Fig. 4a, Supplementary Table S2).

In this study, we performed a comprehensive structural analysis of the HU protein from *Spiroplasma melliferum* KC3 coupled with DSC experiments and revealed the key structural factors responsible for high thermotolerance of the protein. The non-conserved Phe14 and Phe29 residues contribute to strengthening the hydrophobic dimer contact, the semi-conserved Phe31 is responsible for the absence of the internal cavity at the dimer interface, and the Lys35 and Asn92 residues are involved in the formation of unique hydrogen bonds between the monomers. These factors differ from those reported earlier for HU proteins from the thermophilic bacteria *B. stearothermophilus* and *T. maritima*. Our findings confirm the well-known fact that proteins can feature diverse mechanisms that lead to increased thermal stability^33^.

## Methods

### Gene cloning, protein purification, X-ray crystallography, and structure determination

The expression, purification, and crystallization of HUSpm and the X-ray experimental methods have been described earlier^32^. Briefly, the hup2 gene was amplified from the genomic DNA of *S. melliferum* by PCR with the primers (HUSpm.F 5′-GGTGTACATATGTCAAAAAAAGAACTAGC-3′ and HUSpm.R 5′-CTTTCGGAATTCTTAAT; the Nde1 and EcoR1 restriction sites are underlined). The Nde1 and EcoR1 digestion was followed by ligation of the PCR product into the pET-21d cloning vector (Novagen, Darmstadt, Germany) modified as described earlier^32^. The construct was verified by sequencing and transformed into *E. coli* BL21-CodonPlus (DE3)-RIPL competent cells (Stratagene, La Jolla, USA). The culture was grown in an LB/ampicillin medium at 37 °C until the OD600 value of 0.8 was reached, and the expression of HUSpm fused at the N-terminus with 6xHisTev-tag was induced with 1 mM IPTG. After incubation for 18 h at 25 °C, the cells were harvested by centrifugation and the recombinant protein was purified by Ni–NTA affinity chromatography and digested with TEV-protease. After removal of 6xHisTev-tag using a second run of Ni–NTA affinity chromatography, HUSpm was subjected to final purification and buffer exchange by size-exclusion chromatography.

The protein (14 mg/ml in 20 mM Tris HCl, pH 8.0, supplemented with 200 mM NaCl and 5% glycerol) was crystallized at 4 °C using the hanging-drop vapour diffusion method and a reservoir solution composed of 0.1 M Tris HCl, pH 8.0, 35% v/v PEG 400, and 5% glycerol. The mother liquor containing 15% glycerol was used as a cryoprotectant. Crystals were then flash-cooled at 100 K in liquid nitrogen. The X-ray diffraction data were collected at the Belok beamline of the NRC Kurchatov Institute (Moscow, Russian Federation) at a wavelength of 0.984 Å using a MARCCD detector. Crystals of HUSpm belong to the C2 space group. The structure was solved by the molecular replacement method and refined to 1.36 Å resolution. The structural data were deposited in the RCSB Protein Data Bank (entry code 4N1V)^32^.

Due to the poor correlation coefficient (CC_1/2_) and low completeness in the highest shell, the data were reprocessed with the iMosflm program^34^ and the resolution was cut to 1.4 Å. The structure was solved with the MOLREP program^35^ using the structure of the protein determined earlier as a starting model (the solvent was excluded)^32^. The structure was refined with the CCP4 suite^36^. Visual inspection of electron density maps and the manual rebuilding of the model were carried out with the COOT interactive graphics program^37^. The final model comprises 95 residues (including two residues of the N-terminal non-cleaved Gly-His fragment), 124 water molecules, and one sodium ion. The Pro65 residue and the C-terminal Asn94 residue were not observed in electron density maps, likely due to the high mobility of these residues. All stereochemical parameters for side-chain and main-chain atoms were within acceptable limits, with the φ –ψ values of the residues being in the most favoured (99%) or allowed (1%) regions of the Ramachandran plots. Data collection and refinement statistics are given in Table 4.

### Mutant production

Easy single-primer site-directed mutagenesis was performed as described^38^ with minor modifications to make point mutations listed in Supplementary Table S2. The synthetic oligonucleotide primers designed to switch amino acids (one primer for each mutant) and those designed for the selection of mutant clones are listed in the Supplementary Table S3. Eighteen cycles of PCR were performed on the template of the HUSpm-expressing plasmid^32^ using the Tersus Plus PCR kit (Evrogen, Moscow, Russia) according to the manufacturer’s recommendations. The PCR products were treated with DpnI endonuclease (Thermo Fisher Scientific, Massachusetts, United States), which digested the parental DNA template, and then transformed into *E. coli* Match1 competent cells. The mutant clones were selected by PCR performed directly on colonies using Taq DNA polymerase (Evrogen, Moscow Russia) and check primers (Supplementary Table S3) with an appropriate T7 universal primer. Plasmid DNA purified from mutant clones was sequenced to ensure the absence of random mutations associated with PCR. The expression and purification of mutant proteins was performed in the same way as described for HUSpm^32^. The purity of the mutants was estimated by SDS–PAGE with Coomassie staining, and the protein concentration was measured using the Bicinchoninic Acid Protein Assay Kit (Sigma-Aldrich, St. Louis, USA).

### Differential scanning calorimetry

The excess heat capacity of the denaturation of HUSpm (WT) and its mutants was measured on a DASM-4M differential adiabatic scanning microcalorimeter with 467 μl capillary cells under a constant pressure of 2.2 atm at a heating rate of 1 K/min.

HUSpm, which was dissolved in a 10 mM sodium phosphate buffer, pH 7.4, containing 0.2 or 1.0 M NaCl to a concentration of 4.5 or 1 mg/ml, was used to examine the effect of the ionic strength and protein concentration on thermal denaturation. To evaluate the role of single amino acid residues in the thermal stability of HUSpm, the mutants and the wild-type protein reference control were dissolved in a 10 mM sodium phosphate buffer, pH 7.4, containing 0.2 M NaCl to a concentration of 2.0 mg/ml. This protein concentration was sufficient for obtaining good quality DSC data for all the proteins under study with minimal protein consumption.

### Structure analysis and validation

The visual inspection of the structure model was carried out with the COOT and Pymol (The PyMOL Molecular Graphics System, Version 1.2r3pre, Schrödinger, LLC). The multiple sequence alignment was performed with Clustal Omega^39^. The structures of the monomers were compared using the PDBeFOLD program^40^. The contacts were analysed using the PDBePISA^41^ and WHATIF servers^42^. The free energy of solvation upon the formation of the dimer was estimated with PDBePISA. Verification of the structure was made with MolProbity^43^.

## Additional Information

**How to cite this article:** Boyko, K. *et al*. Structural basis of the high thermal stability of the histone-like HU protein from the mollicute *Spiroplasma melliferum* KC3. *Sci. Rep.* **6**, 36366; doi: 10.1038/srep36366 (2016).

**Publisher’s note**: Springer Nature remains neutral with regard to jurisdictional claims in published maps and institutional affiliations.

## Supplementary Material

Supplementary Information

## Figures and Tables

**Table 1:** Hydrogen bonds involved in the formation of the dimer contact region in HUSpm (according to PISA and WHATIF).

	A.a 1 position	A.a 1 name	Atom 1	Distance, Å	A.a 2 name	A.a 2 position	Atom 2
1	**−1**	**GLY**	**O**	**2**.**9**	**GLU**	**43**	**N**
2	1	MET	N	2.8	GLU	43	O
3	1	MET	O	2.8	SER	45	N
4	3	LYS	N	3.0	SER	45	O
5	**35**	**LYS**	**NZ**	**2**.**7**	**GLY**	**48**	**O**
6	**43**	**GLU**	**N**	**2**.**9**	**GLY**	**−1**	**O**
7	43	GLU	O	2.8	MET	1	N
8	45	SER	N	2.9	MET	1	O
9	45	SER	O	3.0	LYS	3	N
10	**48**	**GLY**	**O**	**2**.**7**	**LYS**	**35**	**NZ**
11	77	LYS	N	2.8	ASN	92	OD1
12	77	LYS	O	2.8	ASN	92	ND2
13	**92**	**ASN**	**OD1**	**2**.**8**	**LYS**	**77**	**N**
14	**92**	**ASN**	**ND2**	**2**.**8**	**LYS**	**77**	**O**

Hydrogen bonds with donor-to-acceptor distances shorter than 3.5 Å are listed. Hydrogen bonds unique for HUSpm are given in bold.

**Table 2:** Melting points of HU proteins from mesophilic and thermophilic organisms measured in various conditions.

	Source organism^[reference]^, (PDB code)	T_melt_*, °C	Experimental conditions	
			Protein concentration, mg/ml	Salt concentration, M
1	*Spiroplasma melliferum* KC3, (5CVX)	75.5, 92.2	4.5	0.2
		84.8, 103.3	4.5	1.0
		69.9, 81.0	1.0	0.2
2	*E. coli*^14^ HUα2 (2O97), HUβ2 (4P3V) Huαβ (NA)	40, 57; 26, 59; 41, 62	4.5	0.2
		41, 50; 27, 56; 38, 53	1.0	0.2
		59, 69; 50, 65; 60, 72	1.0	1.0
3	*Bacillus stearothermophilus*^23^, (1HUU)	65.8	0.2	0.05
4	*Bacillus subtilis*^21^, (NA)	46	1.7	0.1
		61	1.9	0.5
		32	0.1	no
		55	0.1	0.5
5	*Bacillus globigii*^21^, (NA)	41	0.05	no
6	*Bacillus caldolyticus*^21^, (NA)	68	0.05	no
7	*Thermotoga maritima*^26^, (1B8Z)	77.5	1.2	0.05
8	*Thermoplasma volcanium*^25^, (NA)	57.1	1.6	0.1
		54.8	0.6	0.1

^*^Two values of Tm are given for proteins with two peaks in the melting curves. NA stands for not available.

**Table 3:** Comparison of dimer contacts in structurally characterized HU proteins with known thermal stability.

	Source organism*	Buried surface area of a monomer at the interface, Å^2^ (%)	Total surface area of a monomer, Å^2^	Hydrogen bonds (shorter than 3.5 Å)	Salt bridges (shorter than 4.0 Å)	Number of residues involved in the interface (hydrophobic) per monomer	Δ^i^G, kcal/mol
1	*Spiroplasma melliferum*	2267.0 (30.2)	7508.3	14	0	52 (35)	−44, 3
2	*Escherichia coli* (HUα2)	1773.3 (32.7)	5419.0	5	6	46 (38)	−35, 2
3	*Escherichia coli* (HUβ2)	1810.9 (34.2)	5300.3	9	4	47 (30)	−32.2
4	*Thermotoga maritima*	1868.9 (33.4)	5595.3	7	5	47 (32)	−40.1
5	*Bacillus stearothermophilus*	1778.4 (27.8)	6404.9	6	4	44 (33)	−41.4

The numbers of hydrogen bonds, salt bridges, and hydrophobic residues at the dimer interface were calculated by WHATIF and manually checked.

^*^The name of the source organism is underlined if the corresponding HU protein has high thermal stability.

**Table 4:** Structure solution and refinement statistics for HUSpm.

PDB code	5L8Z
Data collection statistics*	
Beamline	NRC “Kurchatov Institute” (beamline K4.4 ≪Belok≫)
Detector type	Rayonix SX-165 CCD Detector
Wavelength (Å)	0.984
Data collection software	MarCCD
Space group	C121
Cell dimensions	
*a*, *b*, *c* (Å)	57.0, 39.01, 38.78
α, β, γ (°)	90, 108.36, 90
Resolution range (Å)	31.64-1.40 (1.42-1.40)
R_merge_ (%)	4.9 (27.9)
<I>/<σ(I)>	13.5 (3.5)
Completeness	95.4 (96.8)
Redundancy	3.5 (3.5)
Refinement statistics**	
No. reflections	14567
R_work/_R_free._	18.0/21.1
Number of non-H atoms	
Protein	719
Ligans/ion	1
Water	124
Average B-factor	15.0
Protein	17.18
Ligands/ion	17.06
Water	27.13
R.m.s deviations	
Bond length (Å)	0.019
Bond angle (°)	2.229
Ramachandran favoured (%)	98.9
Ramachandran allowed (%)	1.1
Ramachandran outliers (%)	0.0
MolProbity score	1.59

^*^The highest resolution shell is shown in parentheses. R_merge_ = Σ_hkl_Σ_j_ |I_hkl,j _− <I_hkl_>)/Σ_hkl_Σ_j_ <I_hkl,j_>.

^**^R = {Σ||F_obs_|_ _− |F_calc_||}/Σ|F_obs_|, where |F_obs_| and |F_calc_| are observed and calculated structure factor amplitudes, respectively. R_free_ was calculated for 5% randomly selected reflections of data sets that were not used in the refinement. R_work_ was calculated with remaining reflections. MolProbity score combines the clashscore, rotamer, and Ramachandran evaluations into a single score, normalized to be on the same scale as X-ray resolution.
