# Supplementary material for: Structural basis of the high thermal stability of the histone-like HU protein from the mollicute Spiroplasma melliferum KC3
Source: Sci Rep. 2016 Nov 3;6:36366. doi: 10.1038/srep36366 (PMC5093408; doi:10.1038/srep36366)

SUPPLEMENTARY MATERIALS

Structural basis of the high thermal stability of the histone-like HU protein from the mollicute *Spiroplasma melliferum* KC3

Konstantin Boyko, Tatiana Rakitina, Dmitry Korzhenevskiy, Anna Vlaskina, Yuliya Agapova, Dmitry Kamashev, Sergey Kleymenov, and Vladimir Popov

Supplementary Table S1. Superposition of the three-dimensional structure of the HUSpm monomer with that of the structurally characterized HU proteins from different bacteria. Only backbone atoms were superposed.

	Name of source organism	PDB code	Z-score	RMSD*, A	Number of aligned residues	Total number of residues
1	*Bacillus stearothermophilus*	1HUU	9.6	1.0	80	90
2	*Bacillus anthracis*	3RHI	9.1	1.57	84	93
3	*Thermotoga maritima*	1B8Z	8.6	1.1	71	90
4	*E. coli* (HUα2)	2O97	8.4	1.09	70	90
5	*E. coli* (HUβ2)	4P3V	8.7	1.19	72	72
6	*Anabaena sp.*	1P71	8.0	1.64	81	94
7	*Borrelia burgdorferi*	2NP2	7.5	1.8	82	108
8	*Staphylococcus aureus*	4QJU	8.9	1.5	77	90
9	*Mycobacterium tuberculosis*	4PT4	9.6	1.1	78	97
10	*Streptococcus mutans*	5FBM	9.6	1.16	82	83

*RMSD - Root mean square deviation.

Supplementary Table S2. Effects of mutations on melting temperatures of HUSpm measured by DSC in the following experimental conditions: 2.0 mg/ml of protein and 0.2 M NaCl.

**Mutation**	WT	F14A	F29A	V17T	F31L	K35T	N92K
**T_{melt}^{*}, °C**	74.5, 87.3	63.0	45.2, 59.2	74.2	64.2	49.7, 77.1	70.7

*In case of two peaks on DSC curve, two temperatures are indicated.

Supplementary Table S3. List of synthetic oligonucleotide primers designed to switch amino acids and check primers for selection of mutant clones.

Name	Sequence (from 5’ to 3’)	Tm, °C*
F14A.F	GCCGAAAAA**GCT**ACTGATGTCTTATCAA<br>Check14.R: TTGATAAGACATCAGT**AGC**	40
F29A.R	ATGATC**G**AAAACA**GC**ATTTGTAATTTCTTCTGC<br>Check29.F: GCAGAAGAAATTACAAAT**GC**	44
V17T.F	GAAAAATTTACTGAT**ACC**TTATCAAAAACACATGC<br>Check17.R: GCATGTGTTTTTGATAA**GGT**	42
F31L.F	ACAAATTTTGTT**C**T**C**GATCATATTAAAAAAGC<br>Check31.R: GCTTTTTTAATATGATC**G**A**G**	40
K35T.F	GATCATATTA**CC**AAAGCTTTAGTTGCTGG<br>Check35.R: CCAGCAACTAAAGCTTT**GG**	47
N92K.F	AACTGATTTAAA**G**AATAATTAAGAATTCGAGC<br>Check92.R: CTCGAATTCTTAATTATT**C**	46

Mutations are shown in bold. Sequences following the mutations (3’ arms of primers) used for calculation of melting temperatures (Tm) are underlined. *Tm – was calculated by salt adjusted methods (http://biotools.nubic.northwestern.edu/OligoCalc.html) and used as the annealing temperature during PCR-based mutagenesis.

Supplementary Figure S1. Superposition of monomers of HUSpm (shown in green), *E. coli* HU protein (magenta), *T .maritima* HU protein (blue), *B. stearothermophilus* HU protein (red), and *Anabaena* PCC7120 HU protein (grey).

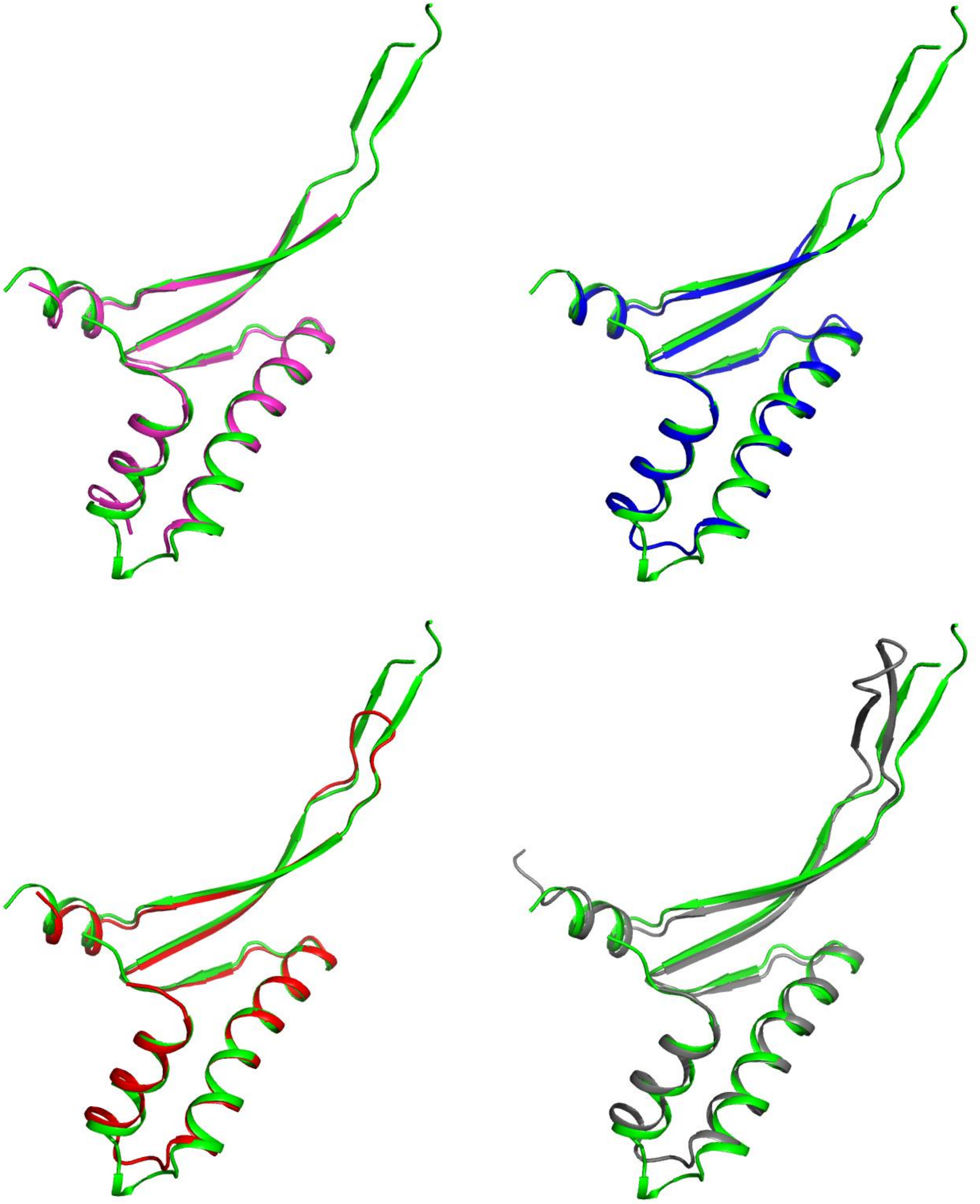

Supplement: Supplementary Information [file srep36366-s1.pdf]
